# The Associations between Diet and Socioeconomic Disparities and the Intestinal Microbiome in Preadolescence

**DOI:** 10.3390/nu13082645

**Published:** 2021-07-30

**Authors:** Yelena Lapidot, Leah Reshef, Rebecca Goldsmith, Wasef Na’amnih, Eias Kassem, Asher Ornoy, Uri Gophna, Khitam Muhsen

**Affiliations:** 1 Department of Epidemiology and Preventive Medicine, School of Public Health, The Sackler Faculty, Tel Aviv University, Ramat Aviv, Tel Aviv 6139001, Israel; lena.lapidot@gmail.com (Y.L.); wasef25@yahoo.com (W.N.); 2 The Shmunis School of Biomedicine and Cancer Research Faculty of Life Sciences, Tel Aviv University, Tel Aviv 6139001, Israel; leahfa@gmail.com (L.R.); microevol@gmail.com (U.G.); 3 Nutrition Division, Ministry of Health, Jerusalem 9101002, Israel; gorebecca@gmail.com; 4 Department of Pediatrics, Hillel Yaffe Medical Center, Hadera 3810101, Israel; EiasK@hy.health.gov.il; 5 Adelson School of Medicine, Ariel University, Ariel 4077625, Israel; asher.ornoy@mail.huji.ac.il; 6 Laboratory of Teratology, Department of Medical Neurobiology, The Hebrew University Hadassah Medical School, Jerusalem 9112002, Israel

**Keywords:** microbiome, dietary intake, school age, socioeconomic status, obesity

## Abstract

The intestinal microbiome continues to shift and develop throughout youth and could play a pivotal role in health and wellbeing throughout adulthood. Environmental and interpersonal determinants are strong mediators of the intestinal microbiome during the rapid growth period of preadolescence. We aim to delineate associations between the gut microbiome composition, body mass index (BMI), dietary intake and socioeconomic status (SES) in a cohort of ethnically homogenous preadolescents. This cohort included 139 Arab children aged 10–12 years, from varying socioeconomic strata. Dietary intake was assessed using the 24-h recall method. The intestinal microbiome was analyzed using 16S rRNA gene amplicon sequencing. Microbial composition was associated with SES, showing an overrepresentation of *Prevotella* and *Eubacterium* in children with lower SES. Higher BMI was associated with lower microbial diversity and altered taxonomic composition, including higher levels of *Collinsella*, especially among participants from lower SES. Intake of polyunsaturated fatty acids was the strongest predictor of bacterial alterations, including an independent association with *Lachnobacterium* and *Lactobacillus*. This study demonstrates that the intestinal microbiome in preadolescents is associated with socioeconomic determinants, BMI and dietary intake, specifically with higher consumption of polyunsaturated fatty acids. Thus, tailored interventions during these crucial years have the potential to improve health disparities throughout the lifespan.

## 1. Introduction

The intestinal microbiome is interconnected with health and wellbeing throughout the life cycle, especially during the early years of life [1], and during the rapid growth periods in later childhood [2]. Recent studies have established that the microbiome continues to shift and develop throughout youth and could play a pivotal role in the development or prevention of various conditions [3], including manifestations in the gut–brain axis [4,5], the gut–liver axis [6,7], immune function [8] and various metabolic diseases [9,10]. Thus, it is crucial to better understand the relationship between the developing child’s microbiome and various environmental exposures, including nutritional status and socioeconomic determinants.

Diet is a key regulator of microbiome structure and function across the lifespan [11]. Microbial colonization in the first years of life has been addressed; however, studies during later childhood and adolescence are sparse [12]. Moreover, socioeconomic status (SES) is a strong determinant of both dietary-associated health disparities [13,14] and intestinal microbiome [15,16,17]. Nonetheless, the relationships of socioeconomic factors, dietary intake and the intestinal microbiome remain elusive, especially during the first two decades of life. This crucial developmental period represents a unique opportunity to beneficially manipulate the microbiome through dietary interventions, which in turn has the potential to affect both short- and long-term health outcomes.

Herein, we aim to delineate the associations of SES and dietary intake on the intestinal microbiome composition of healthy pre-adolescents.

## 2. Materials and Methods

### 2.1. Study Population and Design

This study was conducted in two Arab villages (referred to as villages A and B) in northern Israel, located ~10 km apart in the Hadera subdistrict. The Arab population in Israel is in transition, marked by consistent improvement in educational attainment and in health indicators.

During 2017, 12,900 residents lived in village A and 14,400 in village B [18,19]. The villages were specifically selected to represent various socioeconomic strata. The SES ranks of the villages ranged from 2 to 3 on a scale of 1–10; the highest rank is 10, i.e., a higher rank represents a better SES [20]. The villages are connected to piped water and sanitation infrastructure, and are connected to telephone, internet and cable television networks. The villages have primary care clinics managed by the health maintenance organizations, and mother and child health clinics which provide preventive services.

The traditional diet in the Arab population is based on the Mediterranean diet, including large portions of vegetables, fruit, and olive oil [21]. However, shifts towards Westernized diets are evident, including manufactured food, sweets, and soft drinks [22].

The original cohort comprised 233 children: 134 (57.5%) from the lower SES village (B) and 99 (42.5%) from the higher SES village (A). The children were recruited at age 1–9 weeks during 2007. The cohort has been described elsewhere [23]. Inclusion criteria were: (1) a singleton birth; (2) no prenatal/perinatal complications; (3) no birth defects or diseases that might affect growth; (4) birth weight > 2 kilogram (kg); (5) gestational age at birth > 34 weeks.

During 2017–2019, a follow-up examination was conducted of the same children, aged 10–12 years. Of the 233 children in the original cohort, one child died due to a road accident, two children with developmental delay and special needs were excluded from the current analysis, and 24 were not located. Overall, 207 (88.8%) families were successfully contacted. Of them, 18 (8.7%) refused to participate, 15 (7.2%) initially consented but subsequently withdrew their consent, and 174 (84.1%) agreed to participate in the study. Of the 174 children, 149 completed both the dietary intake and anthropometric measurements and 139 provided a stool sample for microbiome analysis. Compliance to take part in the study was 75% (Supplementary Figure S1).

### 2.2. Data Collection and Definition of the Study Variables

Parents who provided informed consent were interviewed (face-to-face) in Arabic to collect information on the children’s health status and dietary intake at school age.

### 2.3. Dietary Intake Questionnaire

Information was collected on dietary intake using the 24-h food recall method [22]. Twenty-four-hour dietary recall interviews (multiple pass) were performed in-person in Arabic with the participants and their mothers, according to a protocol of the Israeli Ministry of Health (MOH). Using three passes, the interviewer asked the participant to report his/her food intake during the 24 h preceding the interview.

The first pass was a quick list, in which the participants were asked to recall all that they had eaten and drunk during the 24 h prior to the interview. The second pass was a detailed description of all the items mentioned in the first pass (quick list). In the third pass, the interviewer assessed additional items of foods and beverages that might have been missed in the earlier passes. The Arabic version of the MOH “*Food and Food Quantities Guide*” was used to assist the participants in identifying foods and to quantify food during the dietary recall. The guide was designed to enhance the accuracy of dietary intake assessment and comprises pictures of common Israeli foods and comprehensive questions about food. The interviewers underwent standard training on performing these interviews, including lectures, exercises, supervised simulations, a real-life exercise, and feedback. Quality control on the collected data was performed to assess completeness and coherence. Feedback was provided to the interviewers as needed.

### 2.4. The Dependent Variable

The intestinal microbiome at school age was characterized by 16SrRNA sequencing and further statistical analysis, as described below.

### 2.5. The Main Independent Variable

Dietary intake at school age was classified based on the 24-h food recall questionnaires as described above. Data from these questionnaires were entered to the MOH’s computerized dietary analysis program “TZAMERET” and nutrients database, which includes the Israeli national foods. Reports of macronutrient and micronutrient intake were generated. Dietary intake of nutrients was computed as continuous variables and adjusted for the percentage of daily energy intake in Kilocalories (Kcals) and expressed as Kcal as % of daily intake.

### 2.6. Co-Variates

Sociodemographic variables included: village of residence, age at enrollment, sex, number of maternal and paternal schooling years. Household density index was calculated as the number of persons living in the household divided by the number of rooms in the household. We created a composite score using confirmatory factor analysis that included the variables: (a) residential SES rank; (b) number of paternal schooling years; and (c) household crowding index. The analysis was implemented using “Principal Axis” method, including rotation with “varimax” (R package psych). The selected variables were tested with Bartlett’s test of homogeneity of variances (Bartlett’s K-squared = 504.2, df = 2, *p* < 2.2 × 10^−16^) and Kaiser–Meyer–Olkin factor adequacy resulting with adequate scores for all selected variables: village of residence = 0.61; crowding index = 0.7; and number of parental schooling years = 0.63. The newly generated SES score was composed from a combination of the standardized loadings of the above-mentioned variables: residential SES = 0.7; crowding index = 0.5; parental schooling years = 0.6 and was normally distributed. The SES score was analyzed as a continuous variable and as a categorical variable (below and above the median score (6.4), representing low and high SES groups, respectively).

Anthropometric measurements. Measured height and weight were obtained by trained research assistants. Standing height (without shoes) was recorded to the nearest 0.1 centimeter (cm). Weight was measured (with light clothing) to the nearest 0.1 kg, using a calibrated digital scale.

Body mass index Z (BMIZ) score (a continuous variable) was calculated using weight and height measurements as compared to World Health Organization (WHO) data for ages 5–19 years [24]. BMI was calculated as: weight (kg)/height^2^ (meters [m]). The BMIZ score was further analyzed as a categorical variable. Children with BMIZ scores between ≥−2 and <1 standard deviations (SD) compared to the WHO reference population were considered as having normal weight. Children with BMIZ scores between ≥1 SD and ≤2 SD (equivalent to BMI between ≥25 and <30 kg/m^2^ at age 19 years) were classified as overweight, while >2 SD (equivalent to BMI 30 kg/m^2^ at age 19 years) were classified as obese [25,26].

### 2.7. Data Management

Data obtained through interviews and measurements were subjected to quality control checks to assess completeness and consistency and analyzed using IBM SPSS Version 25 (IBM Corp, Armonk, NY, USA). WHO Anthro Plus were used to calculate age-and sex adjusted BMIZ score, compared to the WHO growth standard for populations. The TZAMERET software and database served for entry of data from the 24-h recall questionnaires to generate data on the intake of various nutrients.

### 2.8. Sample Collection, DNA Extraction and Bacterial DNA Amplification

Stool samples were collected using collection plastic cups and transferred on ice to the study laboratory at Tel Aviv University. Samples were divided into two aliquots and stored at −80 °C until testing.

DNA extraction was performed using the Magcore Nucleic Acid Extractor (RBC) and the MagCore DNA Tissue kit according to the manufacturer’s instructions, with the following adaptation: the samples were first transferred to a tube containing beads (Bead Beating Tube Type C (Soil); Geneaid). Buffer GT from the Magcore kit was added to the tubes, and then the tubes were placed in a Bead Beater (BioSpec Products, Inc., Bartlesville, OK, USA) for two minutes. Proteinase K was then added to the samples and the extraction proceeded according to the manufacturer’s protocol for the Magcore DNA Tissue Kit (RBC). DNA concentrations were measured by nanodrop, and 20 ng of DNA was used for 16S library preparation.

We prepared 16S libraries for Illumina sequencing using a two-step PCR protocol. In the first PCR, primers containing tails were used to amplify the v4 region of the 16S rRNA gene. The Access Array primers for Illumina (Fluidigm) are used in the second PCR to add the adaptor and index sequences to each sample. After the second PCR, the reactions are cleaned using Kapa Pure beads (Kapa) to remove unincorporated primers and any primer–dimer produced. The concentration of each library was measured by Qubit (Life Technologies, Carlsbad, CA, USA) using the Denovix ds DNA HS assay (Deonvix) and then the samples were combined in equal amounts into a single pool. The pooled sample was run on the tape station (Agilent, Santa Clara, CA, USA) using a DNA 1000 screen tape to determine the size of the pooled libraries. The pooled library was then loaded on the Illumina Miseq and sequenced using a Miseq v2 Kit (500 cycles) to generate 2 × 250 PE reads. The data were de-multiplexed using Base space, the Illumina cloud software, to generate 2 FASTQ files per sample. The FASTQ files were further analyzed using CLC-bio (Qiagen, Hilden, Germany) to generate abundance and OTU tables.

### 2.9. Statistical Analyses

Quality control analysis of demultiplexed reads was performed using the Deblur [27] workflow using default parameters, following the construction of a phylogenetic tree (mafft-fasttree) and taxonomy assignment with QIIME2 software [28]. The quality process with Deblur uses sequence error profiles to obtain putative error-free sequences, referred to as “sub” operational taxonomic units (s-OTU). Taxonomic composition was assigned to the s-OTUs using a pre-trained Naive Bayes classifier, trained on the Greengenes [29] 13_8 99% OTUs.

Downstream analysis was conducted using R version 4.0. Diversity analysis was calculated at rarefaction depth of 9858. Bacterial α-diversity was estimated using the number of observed OTUs, Shannon’s diversity and Fisher’s diversity indexes and compared across independent variables using the Kruskal–Wallis test for categorical and Welsh ANOVA for continuous variables. β-diversity was calculated using the phylogenetic Weighted and Unweighted Unifrac distances. Permutational multivariate analysis of variance (PERMANOVA) was used to test differences in overall microbiome composition [30] (vegan; adonis2 [31]), implementing a multivariate model with the following covariates: SES, BMI and the reported dietary intake, expressed as percentage of the total daily intake (Kcals). Pairwise comparisons were performed using Dunn’s test, and controlled for false discovery rate (FDR) with the Benjamini–Hochberg method (*p* < 0.05).

Differential abundance analysis was performed using Microbiome Multivariable Associations with Linear Models (MaAsLin2) [32], a multivariable statistical framework for examining potential associations between clinical metadata and potentially high-dimensional microbial multiomics data. MaAsLin2 finds associations between microbial features and complex metadata in population-scale epidemiological studies, allowing us to include host related environmental exposures as diet and SES determinants.

The current analysis was performed after filtering at minimum abundance level of 0.005 and minimum prevalence of 0.2 at the genus taxonomic level. We applied negative binomial models as the chosen analysis method, after normalization of the count data using EdgeR’s Trimmed Mean of M-values (TMM) [33], a technique that uses a weighted trimmed mean of the log expression ratios between samples. The q-value threshold for significance was 0.1, after multiple hypothesis correction using FDR.

Differences in demographics and dietary intake were examined using Wilcoxon rank sum test for continuous variables and the chi square test for categorical variables. Post hoc pairwise comparisons were conducted using the Bonferroni test to correct for multiple comparisons.

### 2.10. Ethical Aspects

The Institution Review Board of Hillel Yaffe Medical Center and the ethics committee of Tel Aviv University approved the study. Written informed consent was obtained from parents of the participants.

## 3. Results

### 3.1. Demographic Characteristics and Dietary Intake

The current analysis included 139 (59% males) children aged 10.8 to 11.7 years (median = 11.42). The BMIZ score ranged from 0.22 to 2.59 (median = 1.61). One-third (33%) of the participants lived in Village A and two-third (67%) in Village B. The composite SES score of the cohort ranged between 4.69 and 7.53 (median = 6.45).

**Figure 1:**
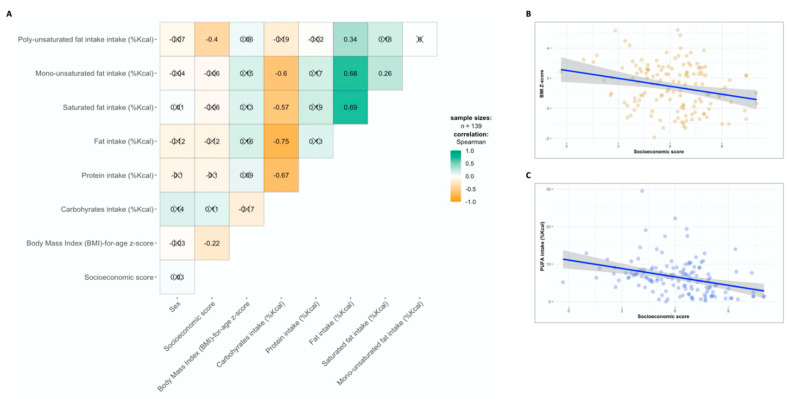
Socio-demographic characteristics and dietary intake (**A**) Correlation matrix of Spearman’s correlation coefficients across the study variables. Positive correlations are colored in green and inverse correlations are colored in orange. (**B**) A scatter plot of the correlation between BMIZ score and SES score. (**C**) A scatter plot of the correlation between intake of PUFAs (%Kcal) and SES score. X in the figure represents non-significant correlation (*p* > 0.05 adjusted for FDR).

The dietary intake was characterized by a relatively high intake of carbohydrates (providing 53% of daily energy), followed by fats (contributing 30% of daily calories) and protein (providing 15% of daily energy intake). Most of the fat intake was monounsaturated fatty acids (MUFAs; 11.4% of daily energy), followed by saturated fats (9.5% of daily energy) and polyunsaturated fatty acids (PUFAs; 6.3% of daily energy). Total dietary fat intake was significantly correlated with consumption of saturated fatty acids (Spearman’s *r* = 0.69, *p* < 0.001), MUFAs (Spearman’s *r* = 0.68, *p* < 0.001) and PUFAs (Spearman’s *r* = 0.34, *p* < 0.001). Carbohydrates intake was inversely correlated with protein intake (Spearman’s *r* = −0.67, *p* < 0.001), fat (Spearman’s *r* = −0.75, *p* < 0.001), saturated fat (Spearman’s *r* = −0.57, *p* < 0.001) and MUFAs (Spearman’s *r* = −0.6, *p* < 0.001), but not with PUFAs intake (Figure 1A).

The BMIZ score was negatively correlated with SES scores (Spearman’s *r* = −0.22, *p* = 0.009; Figure 1B), while PUFAs intake was depleted with increased SES scores (Spearman’s *r* = 0.34, *p* < 0.001; Figure 1C).

The median BMIZ score was significantly higher among participants from the lower SES group (*p* = 0.024), with 65% of participants being overweight or obese in this group, compared to 34% in the higher SES group. The intake of macronutrients was not significantly different between study groups; however, there was a trend of consuming more protein and fat among the low SES group. There was a significantly higher consumption of PUFAs amongst children from lower SES group (*p* = 0.001) (Table 1).

An analysis of the dietary intakes of participants classified as overweight and obese compared to their normal weight counterparts (Supplementary Figure S2), revealed that overweight/obese children consumed less carbohydrates (*p* = 0.025) and a higher percentage of calories from fat (*p* = 0.05), including saturated fatty acids (*p* = 0.105) and MUFAs (*p* = 0.079).

### 3.2. Microbial Diversity and Composition

Intestinal microbiome richness was not associated with SES scores (Shannon’s diversity *p* = 0.31, Observed OTUs *p* = 0.129; Supplementary Figure S3A). The BMIZ score was associated with a decrease in the number of observed OTUs, driven by the decreased richness among obese participants as compared to normal weight and overweight participants, but the association was not statistically significant (*p* = 0.09 for both pairwise comparisons; Supplementary Figure S3B).

**Figure 2:**
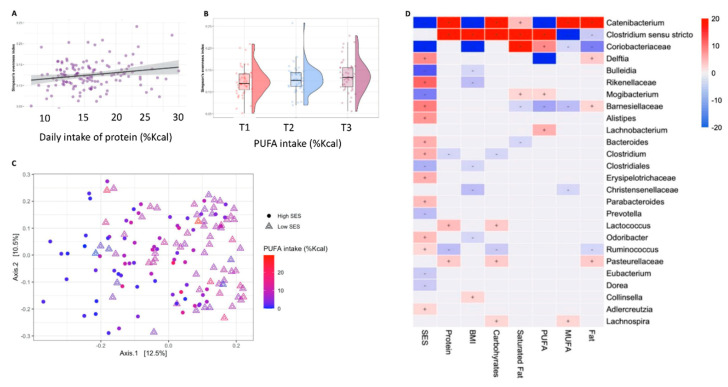
Bacterial diversity and composition. (**A**) A scatter plot of the correlation between alpha diversity, evaluated by Simpson’s evenness index, increased and intake of protein (%Kcal/day). (**B**) Box plot of alpha diversity, evaluated by Simpson’s evenness index according to tertiles consumption of PUFAs (%Kcal/day) (T1 = lowest tertile, T2 =middle tertile, T3 = upper tertile). (**C**) Principal component analysis (PcoA) of the Unweighted UniFrac distance showing a significant separation based on the SES score and intake of PUFAs. (**D**) A heatmap of the multivariable model describing the top 50 associations between the independent variables and bacterial features. Positive associations are colored in red, while inverse associations are colored in blue. The color gradient represents the strength of the association (the effect size), with darker colors representing the stronger associations. The effect size was calculated by the following formula: (−log(qval)*SIGN (coeff)).

There was no significant association between alpha diversity with carbohydrates and fats intake (Shannon’s diversity *p* = 0.63 and *p* = 0.47, Observed OTUs *p* = 0.28 and *p* = 0.35), including saturated fatty acids and MUFAs (Shannon’s diversity *p* = 0.23 and *p* = 0.43, Observed OTUs *p* = 0.35 and *p* = 0.34). The percentage of calories consumed from protein was significantly associated with Simpson’s evenness index (F = 5.38, *p* = 0.02; Figure 2A). The consumption of PUFAs was associated with Simpson’s evenness index, yet this association was not statistically significant (F = 3.35, *p* = 0.069; Figure 2B).

A permutation-based analysis of variance model, which included the BMIZ score, SES score and calorie adjusted intake explained 11% of the intestinal microbial variance (Supplementary Table S1). Both the SES and BMIZ scores were significantly associated with microbial composition, with 5% of the variance explained by SES, and 1.2% by BMIZ (R^2^ = 5%, *p* < 0.001 and R^2^ = 1.2%, *p* = 0.038, respectively; Figure 2C). None of the macronutrients was associated with altered bacterial composition, except for PUFAs intake (R^2^ = 1.2%, *p* = 0.014; Figure 2C).

A multivariable association analysis with linear models revealed specific taxonomic alterations associated with BMIZ score, SES score and dietary intake (Supplementary Table S2; Figure 2D).

The major taxonomic shifts were attributed to the SES, with significantly increased abundance of Barnesiellaceae (*p* < 0.001), Rikenellaceae (*p* < 0.001) including genus *Alistipes* (*p* < 0.001), the genera *Delftia* (*p* = 0.001), *Odoribacter* (*p* = 0.018), *Parabacteroides* (*p* = 0.01), *Bacteroides* (*p* = 0.004), *Clostridium* (*p* = 0.004) and *Ruminococcus* (*p* = 0.072). The genera *Bulleidia* (*p* < 0.001), *Mogibacterium* (*p* < 0.001), *Prevotella* (*p* = 0.017), and *Catenibacterium* (*p* < 0.001) were inversely associated with SES score. However, the increased abundance of *Alistipes*, *Parabacteroides*, *Adlercreutzia* and the family Erysipelotrichaceae, as well as the lower abundance of *Prevotella, Eubacterium* and *Dorea* were attributed to the SES scores alone. The BMIZ score was inversely associated with *Catenibacterium* and Coriobacteriaceae (*p* < 0.001), while the relative abundance of *Clostridium sensu stricto* and *Collinsella* was overrepresented with increased BMIZ score (*p* = 0.001 and *p* = 0.05, respectively). Significant inverse associations were observed between the BMIZ score and *Bulleidia*, Rikenellaceae, Clostridiales, Christensenellaceae and *Odoribacter* (*p* = 0.07, *p* = 0.01, *p* = 0.05, *p* = 0.01, *p* = 0.071, respectively).

Protein and carbohydrates intakes were positively associated with *Catenibacterium* (*p* < 0.001), *Clostridium sensu stricto* (*p* < 0.001), *Lactococcus* (*p* = 0.018, *p* = 0.03, respectively) and members of Pasteurellaceae (*p* = 0.03, *p* = 0.025, respectively), while *Ruminococcus* was depleted with increased daily consumption (*p* = 0.018, *p* = 0.036, respectively). Carbohydrates intake was also associated with the relative abundance of the genus *Lachnospira* (*p* = 0.072).

Fat intake was associated with *Catenibacterium* (*p* < 0.001)*, Delftia* (*p* = 0.031) and members of Barnesiellaceae and Pasteurellaceae (q = 0.085, *p* = 0.047, respectively), while the relative abundance of *Clostridium sensu stricto, Ruminococcus* and members of Coriobacteriaceae were depleted with increased intake (*p* = 0.033, *p* = 0.085, *p* < 0.001, respectively). An association was found between MUFAs intake with *Catenibacterium* and *Lachnospira* (*p* < 0.001, *p* = 0.069, respectively), while the relative abundance of *Clostridium sensu stricto* and members of Coriobacteriaceae, Christensenellaceae and Barnesiellaceae were depleted (*p* < 0.001, *p* = 0.074, *p* = 0.085, *p* = 0.017, respectively). The consumption of saturated fatty acids was associated with *Catenibacterium, Clostridium sensu stricto, Mogibacterium* and members of Coriobacteriaceae (*p* = 0.008, *p* < 0.001, *p* = 0.075, *p* < 0.001, respectively), while members of Barnesiellaceae and genus Bacteroides were depleted (*p* = 0.068, *p* = 0.085, respectively).

The intake of PUFAs was strongly associated with *Clostridium sensu stricto, Lachnobacterium, Mogibacterium* and members of Coriobacteriaceae (*p* < 0.001, *p* = 0.004, *p* = 0.085, *p* < 0.001, respectively), while there was an inverse association with the relative abundance of *Delftia* and members of Barnesiellaceae (*p* < 0.001, *p* = 0.001, respectively).

Since SES was the strongest explanatory variable of intestinal microbiome alterations, and given the differences in age between the two SES groups, we performed a stratified analysis, according to high and low SES groups (as described in the methods section; the low SES group included 69 participants with median SES score of 4.66 and the high SES group included 70 participants with a median SES score of 7.53). This allowed us to further explore the associations of diet, BMI and the intestinal microbiome, independently from the SES effect.

### 3.3. Diet–Microbiome Relationships within the Low-SES Subgroup

**Figure 3:**
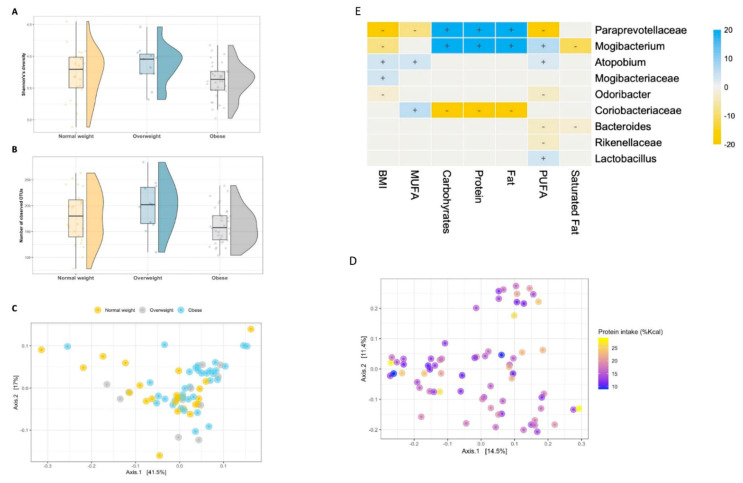
Low socioeconomic status and the intestinal microbiome. Box plots of alpha diversity according to BMIZ score categories. (**A**) Alpha diversity was measured by the Shannon’s diversity index, and (**B**) richness. (**C**) Principal component analysis (PcoA) of the weighted UniFrac showing a significant separation of the BMIZ score categories and (**D**) intake of protein measured by the unweighted UniFrac distance. (**E**) A heatmap of the multivariable model describing the top associations between the independent variables and bacterial features. Positive associations are colored in blue, while inverse associations are colored in yellow. The color gradient represents the strength of the association, with darker colors representing stronger associations. The effect size was calculated by the following formula: (−log(qval)*SIGN (coeff)).

While there was no significant association between dietary intake and bacterial alpha diversity, there was a strong association with BMIZ score. Obese children had significantly lower intestinal microbial diversity (Shannon’s diversity *p* = 0.013, *p* = 0.023; Figure 3A) and richness (*p* = 0.02, *p* = 0.021; Figure 3B) compared to their overweight and normal weight counterparts. BMIZ score was also associated with compositional microbial alterations, as measured by both the weighted and unweighted UniFrac distance matrices (R^2^ = 6%, *p* = 0.023 and R^2^ = 4.1%, *p* = 0.046; Figure 3C). Overall bacterial beta diversity was not significantly associated with macronutrients, except of borderline statistically significant association with protein intake (unweighted UniFrac R^2^ = 2.2%, *p* = 0.049; Figure 3D, Supplementary Table S3). BMI was associated with significant taxonomic shifts (Figure 3E), including a direct association with *Atopobium parvulum* (*p* = 0.03) and members from Mogibacteriaceae (*p* = 0.047), whereas an inverse association was detected between BMIZ-score and *Odoribacter* (*p* = 0.077), *Mogibacterium* (*p* < 0.001) and members from Paraprevotellaceae (*p* < 0.001).

Although the overall bacterial composition was not significantly altered, we found strong taxonomic associations between the intake of several macronutrients and specific features (Supplementary Table S4). Carbohydrates, protein and fat intake were associated with an overrepresentation of *Mogibacterium* (*p* < 0.001) and members of Paraprevotellaceae (*p* < 0.001), while members of Coriobacteriaceae were inversely associated with carbohydrates and protein intake (*p* < 0.001).

Intake of saturated fatty acids was inversely associated with *Mogibacterium* (*p* < 0.001) and *Bacteroides* (*p* = 0.05). MUFAs consumption was positively associated with increased levels of members of Coriobacteriaceae (*p* = 0.004), including *Atopobium parvulum* (*p* = 0.028), and inversely associated with members of Paraprevotellaceae (*p* < 0.001).

Intake of PUFAs was strongly associated with increased relative abundance of *Mogibacterium* (*p* = 0.001), *Lactobacillus* (*p* = 0.039) and *Atopobium parvulum* (*p* = 0.076), while an inverse association was detected with members of Paraprevotellaceae (*p* < 0.001), *Odoribacter* (*p* = 0.02), *Bacteroides* (*p* = 0.035) and members of Rikenellaceae (*p* = 0.036). Interestingly, the relative abundance of *Lactobacillus* and members of Rikenellaceae were independently associated with PUFAs intake among children from the high low group.

### 3.4. Diet–Microbiome Relationships within the High-SES Subgroup

**Figure 4:**
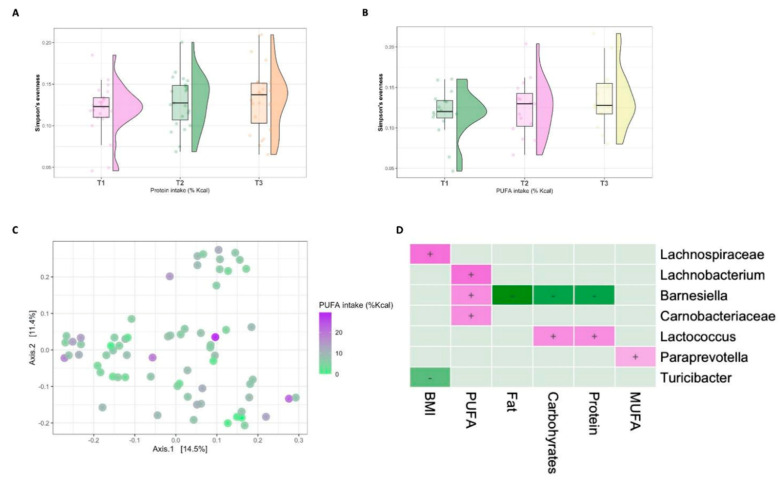
High socioeconomic status and the intestinal microbiome. Box plots of alpha diversity, measured by Simpson’s evenness, according to tertiles intake of (**A**) dietary protein and (**B**) PUFAs (T1 = lowest tertile, T2 = middle tertile, T3 = upper tertile). (**C**) Principal component analysis (PcoA) of Unweighted UniFrac showing the intake of PUFAs among participants from high SES. (**D**) A heatmap of the multivariable model describing the top associations between the independent variables and bacterial features. Positive associations are colored in purple, while inverse associations are colored in green. The color gradient represents the strength of the association observed, with darker colors representing stronger associations. The effect size was calculated by the following formula: (−log(qval)*SIGN (coeff)).

The microbial alpha diversity of children from higher SES was significantly associated with intake of protein (Simpson’s evenness *p* = 0.03; Figure 4A) and PUFAs (Simpson’s evenness 0.042; Figure 4B). There were no significant associations with other macronutrients nor with BMIZ score.

Intake of dietary PUFAs was associated with significant changes of bacterial composition, detected by the unweighted UniFrac (R^2^ = 3.7%, *p* = 0.001; Figure 4C), but not with weighted UniFrac (Supplementary Table S5). Although there were no other significant associations between the variables of interest and global beta diversity, multivariate analysis revealed several features strongly associated with BMIZ score and dietary intake (Figure 4D, Supplementary Table S6).

In line with the significantly altered beta diversity, the strongest taxonomic shifts were associated with PUFAs consumption, including an overrepresentation of *Lachnobacterium* (*p* = 0.014), *Barnesiella* (*p* = 0.035) and members of Carnobacteriaceae (*p* = 0.035). *Lachnobacterium* and Carnobacteriaceae were independently associated with PUFAs in this group. Carbohydrates and protein intakes were positively associated with *Lactococcus* (*p* = 0.039, *p* = 0.035, respectively) and inversely associated with *Barnesiella* (*p* = 0.035). The latter was also inversely associated with fat intake (*p* = 0.015). The consumption of MUFAs was independently associated with increased relative abundance of *Paraprevotella* (*p* = 0.073).

The BMIZ score was positively associated with members of Lachnospiraceae (*p* = 0.014) while inversely associated with *Turicibacter* (*p* = 0.073). These taxonomic alterations were attributed to BMIZ score but not dietary intake.

### 3.5. The Intestinal Microbiome of Overweight/Obese Children

Since overweight and obesity were prevalent in this cohort, with 57% of children classified as overweight or obese, we conducted an independent analysis of microbial alterations, in association with macronutrients intake, among this group.

In line with the relatively higher intake of fats among overweight and obese participants, the macronutrients that were detected by our multivariable analysis as significantly associated with bacterial features were total fat, PUFAs, MUFAs and saturated fatty acids (Supplementary Table S7). Members of Paraprevotellaceae were positively associated with fat intake (*p* = 0.064), but inversely correlated with MUFAs consumption (*p* < 0.001). Members of Barnesiellaceae were inversely correlated with intake of PUFAs and saturated fatty acids (*p* = 0.029 and *p* = 0.005, respectively). The genus *Mogibacterium* was associated with elevated intake of PUFAs and MUFAs (*p* = 0.064), and lastly, *Ruminococcus* was independently negatively associated with MUFAs consumption (*p* = 0.06).

## 4. Discussion

We determined the relative contributions of family SES, dietary intake of macronutrients and BMIZ score in determining intestinal microbiome alterations among pre-adolescents from the same ethnicity. Lower SES was associated with higher BMIZ scores, while participants with greater BMI showed a trend of consuming diets with a higher percentage of fat, including saturated fatty acids and MUFAs. Importantly, we demonstrated that the intestinal microbiome in later childhood is strongly affected by the interplay between various environmental and interpersonal determinants, including SES, BMI and diet.

A growing body of evidence showed that the intestinal microbiome plays a pivotal role in various health phenotypes and diseases [34,35], including the modulation of immunity [36], cardiometabolic manifestations, such as obesity and metabolic syndrome [37,38], liver diseases [7], hyperlipidemia and atherosclerosis [39], chronic kidney disease [40], and even has a relation to neurodevelopmental conditions, via the gut–brain axis [41]. Great interest has emerged in delineating the possible effect of personalized alterations of the microbiome as a means of prevention or improvement of health and wellbeing. Indeed, several studies showed that environmental interventions, including diet, antibiotics and probiotics rapidly influence the host gut microbiome [11,39]. While the available evidence in humans remains descriptive, two recently published studies in well-established mouse models showed a possible causal relationship between environmental dietary (high fiber) and probiotic interventions with the amelioration of the progression of chronic kidney disease [40] and hyperlipidemia [42]. Nevertheless, a gap remains in the understanding of long-term dietary habits and specific nutrients on the host microbiome and long-term health outcomes.

In this cohort, the strongest environmental determinant was SES, which was significantly correlated with both BMIZ scores and gut microbial shifts. SES score explained the largest proportion of variance in this cohort, pointing towards qualitative and quantitative alterations of microbial community composition. Moreover, SES was strongly associated with the highest number of differentially abundant features, including a strong inverse independent association with genera *Prevotella* and *Eubacterium*.

*Prevotella* is considered an important yet enigmatic key genus of the gut microbiome, being a common human gut microbe that has been both positively and negatively associated with human health. Several studies associate *Prevotella* with inflammatory diseases [43,44,45], insulin resistance and glucose intolerance [46], while other studies have linked *Prevotella* with improved glucose and insulin tolerance in association with dietary fiber, especially in rural communities [47,48], suggesting that the beneficial effects could be diet-dependent. Moreover, a higher prevalence of *Prevotella* has been consistently reported in non-Westernized rural populations [49,50]. Thus, in this cohort, while lower SES could be associated with a more traditional, rural lifestyle, the higher abundance of *Prevotella* cannot be explained by an increased intake of fiber, since lower SES was associated with increased intake of dietary fat, but not a substantial increase in fiber intake. Moreover, SES was directly correlated with the genera *Adlercreutzia* and *Alistipes* that were previously described in association with obesity [51,52,53] and *Dorea*, which has been associated with diabetes mellitus [54]. These findings suggest clustering of intestinal microbial phenotypes related to obesity and diabetes in preadolescents from relatively low SES and imply that targeted microbial alternations might affect the risk of obesity and diabetes.

The BMIZ score was independently associated with further microbial alterations, consistently in the whole cohort and across different SES strata. Community level bacterial alterations were detected by an adjusted PERMANOIVA on the phylogenetic weighted and unweighted UniFrac distances. In line with that, significant taxonomic alterations were found including an overrepresentation of the genus *Collinsella* that was independently associated with higher BMIZ score. Interestingly, the relative abundance of *Collinsella* was shown to be associated with type 2 diabetes mellitus [55] including a positive correlation with circulating insulin levels, rheumatoid arthritis [56], and cholesterol metabolism [57]. Furthermore, gnotobiotic approaches showed that the administration of *Collinsella* reduces the expression of tight junction proteins in enterocytes and stimulates gut leakage [56], which are features associated with metabolic endotoxemia [58]. Although the causal mechanisms by which *Collinsella* affects the host metabolism remain elusive, a recent study identified that lower dietary fiber intake is possibly associated with elevated abundance of this genus [59]. Similarly, in this cohort, consumption of carbohydrates and fiber were lower among overweight and obese children than those with normal weight, while intake of fat was elevated.

The intestinal microbiome plays a pivotal role in the maintenance of human metabolic homeostasis and might increase the risk of metabolic and obesity-related complications [60]. Although there is a lack of consensus on the obese-type microbiome configuration, taxonomic and functional alterations, including reduced biodiversity, have been suggested to contribute to the pathogenesis of obesity in both humans and animal models [10]. The altered microbial profile occurring in obese people is considered as an extreme deviation from the microbiota–host mutualism, resulting from the response to a high-fat, high-sugar diet [10]. Excess food intake, especially high-calorie diets, such as high fat and high-sugar diets, is the main determinant of obesity. High-fat diet induced obesity in particular results in perturbations of the gut-microbial composition and the depletion of microbial diversity [38,61,62]. In line with accumulating evidence [10,12,60,63], we show that higher BMIZ score is consistently associated with taxonomic shifts. In the low SES group, where the prevalence of obesity was significantly higher, we detected a strong reduction in bacterial diversity and altered bacterial composition among obese children, in line with accumulating data worldwide.

The effect of the intake of macronutrients on the microbiome was more delicate, with the strongest association detected with consumption of a high fat diet, specifically between intake of PUFAs and intestinal microbiome composition. This association was consistently detected by beta diversity analysis, implying shifts of bacterial community composition. Apart from bacterial features that were simultaneously associated with SES, BMIZ score and other macronutrients intake, PUFAs consumption was independently related with an overrepresentation of *Lachnobacterium*. Children from lower SES consumed significantly higher percentages of daily energy from dietary fat, and in this group, PUFAs intake was associated with the largest number of differentially abundant bacterial features, including an independent positive association with *Lactobacillus*, and inverse association with *Bacteroides* and members of Rikenellaceae. Intake of MUFAs was associated with several bacterial features, none of which was independently associated with MUFA alone, thus, the effect was more delicate and was not associated with overall microbiome composition alterations. This can be explained by the frequent use of olive oil [21], i.e., Oleic acid, in the traditional Arab diet; however, since the intake of MUFAs was equally distributed across the study groups, the associations with the intestinal microbiome were weaker.

The consumption of PUFAs has been widely investigated in relation to cardiovascular diseases, and excessive intake of dietary PUFAs, especially omega-6 fatty acids, such as linoleic acids, and imbalances of the omega-6/omega-3 ratio contribute to metabolic disease along with chronic inflammation [64]. In this cohort, most PUFAs are derived from the intake of foods rich in linoleic acid, including safflower oil, meat, poultry and eggs and limited intake of food sources of linolenic acid, which can be partly explained by the relatively low intake of fish among Arab adolescents [22]. These results are important considering the high burden of heart disease, stroke and diabetes among Arabs in Israel, in addition to high prevalence of obesity and low physical activity [22,65]. Tailored nutritional education programs in this group can be beneficial in treatment and prevention of childhood obesity, thereby aiding in the prevention of cardiometabolic diseases in adulthood.

Simultaneously, high fat diets and nutritional PUFAs have recently been the focus of attention in association with the intestinal microbiome, independently from obesity [62,64]. Intestinal bacteria mediate saturation of PUFAs derived from dietary fat as a detoxifying mechanism in the gastrointestinal tract. To date, various gut microbes have been identified as producing PUFAs-derived intermediate metabolites, specifically and in line with our findings, *Lactobacillus* [66,67]; however, an integrated understanding of the effects of various PUFAs metabolites on host physiology remains elusive, especially during childhood, an important window of opportunity to establish long-lasting health and well-being.

The strengths of the current study include a homogenous cohort of participants from the same ethnic group and geographic location, thereby minimizing the microbial variations attributed to ethnic origin and cultural lifestyle habits. Moreover, our multivariable analysis encompassed environmental determinants, i.e., SES and anthropometric measurements and an in-depth dietary analysis, in an understudied age group. Thus, our results provide new evidence in the field of child growth and development, diet and the intestinal microbiome. Moreover, all the data collected in this study were obtained by trained professionals, thus minimizing the potential for collection errors. Our study has limitations. First, the dietary data were collected using the 24-h recall, which is subject to recall bias and depends on memory, cooperation, and communication ability. We attempted to overcome this by the recruitment of trained, Arabic speaking interviewers, provided standardized training and quality control, and interviewed child–mother pairs to collect accurate information on dietary intake. Moreover, although this cohort focused on preadolescents in the relatively narrow age range of 10–12 years, there were significant differences in the median age between the two SES groups. Our stratified analysis by SES group address this limitation since age is an important potential modifier of the intestinal microbiome. Given the cross-sectional design of our study, we cannot determine the direction of the associations between SES, obesity, and microbiome. More studies are needed to prospectively assess the alterations of the intestinal microbiome with the child’s growth, with close monitoring of changes in diet and BMI. Our findings emphasize the need to expand research in the field of child health from a narrow focus on the first two to three years of life to a wider holistic approach that embraces the varying needs across the lifespan [2,12,68].

## 5. Conclusions

The intestinal microbiome during the crucial developmental window of early preadolescence is profoundly affected by the child’s environment, i.e., SES. Moreover, BMI and dietary habits have an additional, more delicate, yet significant effect on microbial diversity and composition. The effect of macronutrients, and especially PUFAs, was detected even though the cohort consisted of a relatively homogenous group, in terms of culturally determined dietary habits.

## Figures and Tables

**Table 1:** Demographic characteristics and dietary intake of the study participants.

		Socioeconomic Status		
Characteristic	Overall, N = 139 ^a^	High SES, N = 70 ^a^	Low SES, N = 69 ^a^	*p*-Value ^b,c^
Age	11.42 (10.83, 11.67)	11.04 (10.67, 11.50)	11.58 (11.42, 11.75)	<0.001
Sex				0.9
Female	57 (41%)	29 (41%)	28 (41%)	
Male	82 (59%)	41 (59%)	41 (59%)	
Socioeconomic score	6.45 (4.69, 7.53)	7.53 (6.89, 8.13)	4.66 (3.83, 5.49)	<0.001
Village				<0.001
Village A	46 (33%)	37 (53%)	9 (13%)	
Village B	93 (67%)	33 (47%)	60 (87%)	
Household crowding	1.50 (1.15, 2.00)	1.20 (1.00, 1.50)	2.00 (1.50, 2.33)	<0.001
Body Mass Index Z (BMIZ) score	1.61 (0.22, 2.59)	0.92 (−0.13, 2.27)	1.98 (0.68, 2.59)	0.024
BMIZ score classification				0.14
Normal weight	60 (43%)	36 (51%)	24 (35%)	
Overweight	19 (14%)	8 (11%)	11 (16%)	
Obese	60 (43%)	26 (37%)	34 (49%)	
Carbohydrates intake (%Kcal)	53 (48, 57)	53 (49, 57)	51 (47, 57)	0.3
Protein intake (%Kcal)	15.0 (12.2, 18.6)	14.4 (11.8, 18.6)	16.0 (13.0, 18.5)	0.14
Fat intake (%Kcal)	31 (27, 35)	30 (26, 34)	31 (27, 35)	0.4
Saturated fat intake (%Kcal)	9.5 (7.5, 12.0)	9.5 (7.4, 12.3)	9.8 (7.7, 11.7)	0.8
Mono-unsaturated fat intake (%Kcal)	11.4 (9.2, 13.3)	11.0 (9.1, 13.2)	11.7 (9.4, 13.3)	0.5
Poly-unsaturated fat intake (%Kcal)	6.3 (3.2, 8.6)	4.1 (1.6, 7.9)	7.1 (5.5, 9.0)	0.001

^a^ Data presented are median and interquartile range unless specified otherwise. ^b^ *p* Value was obtained by Mann Whitney test for continuous variables and ^c^ Pearson’s Chi square test for categorical variables.

## Data Availability

Individual level data from this study cannot be made publicly available due to legal restrictions.

## Supplementary Materials

The following are available online at https://www.mdpi.com/article/10.3390/nu13082645/s1. Supplementary Figure S1: Flow chart of enrollment and data collection. Supplementary Figure S2: Dietary intake in normal weight compared to overweight and obese participants. Children that were overweight/obese (combined in the figure as overweight) consumed (**A**) significantly less carbohydrates compared to their normal weight counterparts (*p* = 0.025); (**B**) a relatively similar percentage of calories from protein (*p* = 0.275); (**C**) a higher percentage of calories from fat (*p* = 0.050); (**D**) saturated fat (*p* = 0.105); and (**E**) MUFAs (*p* = 0.079). Intake of PUFAs was relatively similar (*p* = 0.536). Supplementary Figure S3: (**A**) No significant association between alpha diversity and socioeconomic scores (Observed OTUs *p* = 0.13). (**B**) A trend of decreased bacterial richness among obese participants compared to their normal weight and overweight counterparts (observed OTUs *p* = 0.09 for both pairwise comparisons). Supplementary Table S1: Bacterial composition in the whole cohort. Supplementary Table S2: Maaslin2 results for the whole cohort. Supplementary Table S3: Bacterial composition in the low SES group. Supplementary Table S4: Maaslin2 results for the low SES group. Supplementary Table S5: Bacterial composition in the high SES group. Supplementary Table S6: Maaslin2 results for the high SES group. Supplementary Table S7: Maaslin2 results for the participants with obesity.
