# Supplementary material for: The Associations between Diet and Socioeconomic Disparities and the Intestinal Microbiome in Preadolescence
Source: Nutrients. 2021 Jul 30;13(8):2645. doi: 10.3390/nu13082645 (PMC8398108; doi:10.3390/nu13082645)
Supplement: Supplementary file 1 [file nutrients-13-02645-s001.zip › nutrients-1302161-supplementary.pdf]

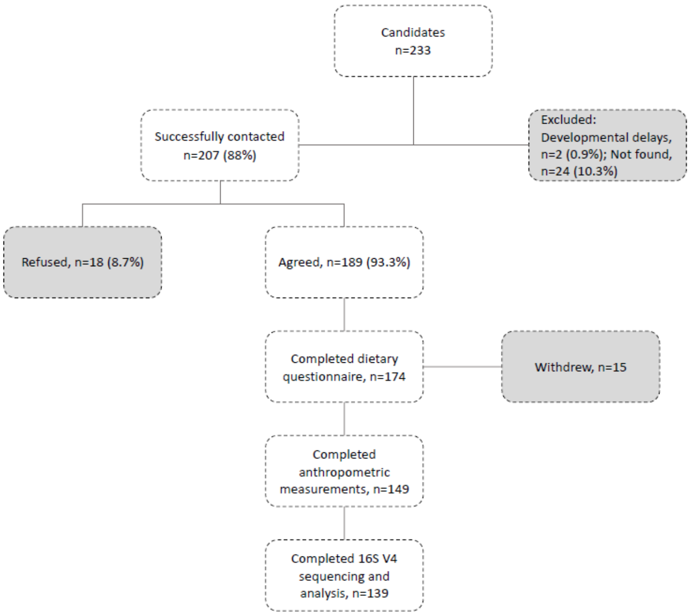

Supplementary Figure S1. Flow chart of enrollment and data collection.

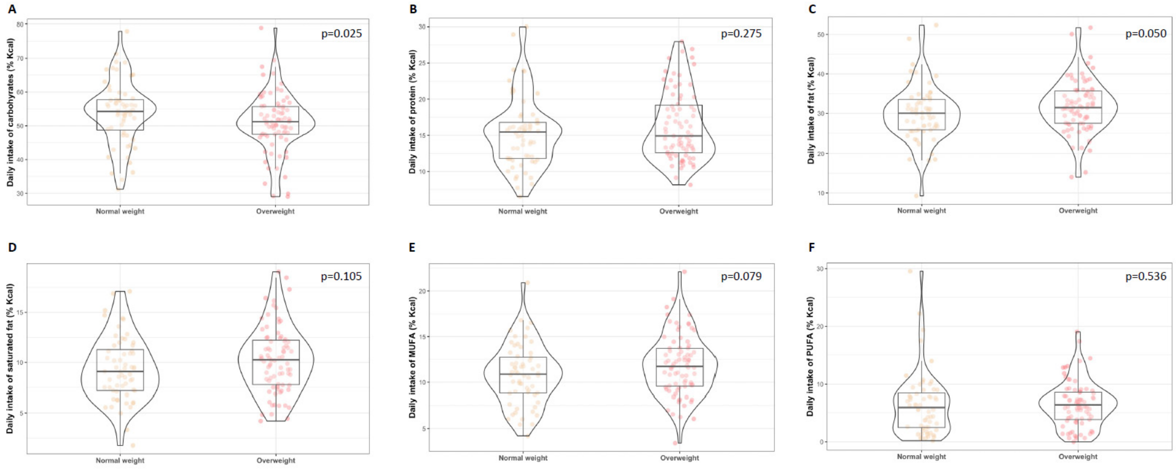

Supplementary Figure S2. Dietary intake in normal weight compared to overweight and obese participants. Children that were overweight/obese consumed (**A**) significantly less carbohydrates compared to their normal weight counterparts (p = 0.027); (**B**) a relatively similar percentage of calories from protein (p = 0.275); (**C**) a higher percentage of calories from fat (p = 0.050); (**D**) saturated fat (p = 0.105); and (**E**) MUFAs (p = 0.079). Intake of PUFAs was relatively similar (p = 0.536).

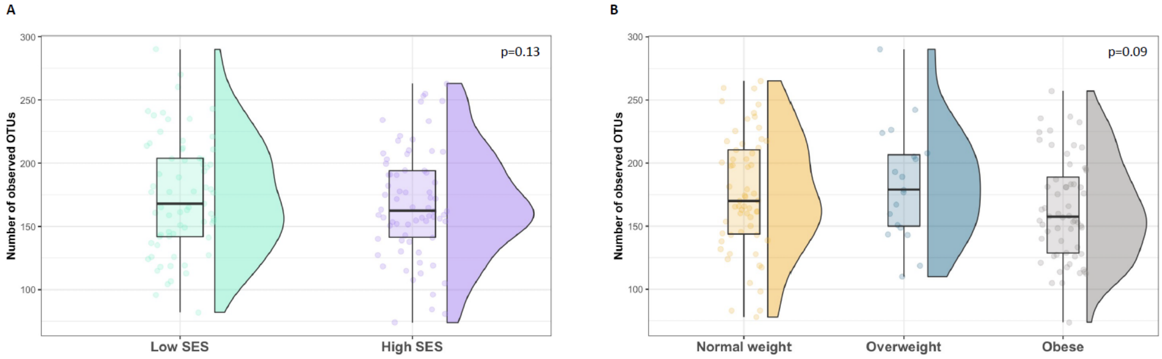

Supplementary Figure S3. (**A**) No significant association between alpha diversity and socioeconomic scores (Observed OTUs p = 0.13). (**B**) A trend of decreased bacterial richness among obese participants compared to their normal weight and overweight counterparts (observed OTUs p = 0.09 for both pairwise comparisons).

Supplementary Table S1. Bacterial composition in the whole cohort.

	Unweighted UniFrac					Weighted UniFrac				
	**Df**	**SumofSqrs**	**R2**	**F**	**p-value**	**Df**	**SumofSqrs**	**R2**	**F**	**p-value**
Body mass index Z-score	1	0.257	0.011	1.558	0.038	1	0.038	0.013	1.914	0.074
Socioeconomic status score	1	1.192	0.05	7.229	< 0.001	1	0.086	0.03	4.268	< 0.001
Fat intake (% Kcal)	1	0.198	0.008	1.204	0.198	1	0.006	0.002	0.317	0.93
Poly unsaturated fatty acids intake (% Kcal)	1	0.286	0.012	1.735	0.014	1	0.017	0.006	0.867	0.479
Mono unsaturated fatty acids intake (% Kcal)	1	0.154	0.006	0.934	0.52	1	0.015	0.005	0.755	0.564
Saturated fatty acids intake (% Kcal)	1	0.205	0.008	1.242	0.155	1	0.004	0.002	0.219	0.972
Protein intake (% Kcal)	1	0.157	0.007	0.952	0.495	1	0.027	0.009	1.325	0.217
Carbohydrates intake (% Kcal)	1	0.146	0.006	0.884	0.611	1	0.012	0.004	0.613	0.705
Residual	130	21.43	0.89			130	2.619	0.927		
Total	138	24.02	1			138	2.826	1		

Adonis2 was implemented, with 1,000 permutations.

Supplementary Table S2. Maaslin2 results for the whole cohort.

**k__Bacteria.p__Actinobacteria.c__Coriobacteriia.o__Coriobacteriales.f__Coriobacteriaceae.g__**	**Socioeconomic status score**	**-0.280**	**0.089**	**134**	**0.002**	**0.022**
k__Bacteria.p__Firmicutes.c__Erysipelotrichi.o__Erysipelotrichales.f__Erysipelotrichaceae.g__Eubacterium	Socioeconomic status score	-0.467	0.161	107	0.004	0.044
k__Bacteria.p__Firmicutes.c__Clostridia.o__Clostridiales.f__Lachnospiraceae.g__Dorea	Socioeconomic status score	-0.129	0.045	139	0.004	0.050
k__Bacteria.p__Actinobacteria.c__Coriobacteriia.o__Coriobacteriales.f__Coriobacteriaceae.g__Adlercreutzia	Socioeconomic status score	0.415	0.151	84	0.006	0.061
k__Bacteria.p__Firmicutes.c__Clostridia.o__Clostridiales.f__Ruminococcaceae.g__Ruminococcus	Socioeconomic status score	0.212	0.079	139	0.008	0.072
k__Bacteria.p__Firmicutes.c__Clostridia.o__Clostridiales.f__Clostridiaceae.g__Clostridium sensu stricto	Body mass index Z-score	0.313	0.009	40	0.000	0.000
k__Bacteria.p__Firmicutes.c__Erysipelotrichi.o__Erysipelotrichales.f__Erysipelotrichaceae.g__Catenibacterium	Body mass index Z-score	-0.094	0.007	58	0.000	0.000
k__Bacteria.p__Actinobacteria.c__Coriobacteriia.o__Coriobacteriales.f__Coriobacteriaceae.__	Body mass index Z-score	-0.171	0.022	51	0.000	0.000
k__Bacteria.p__Bacteroidetes.c__Bacteroidia.o__Bacteroidales.f__Rikenellaceae.__	Body mass index Z-score	-0.741	0.217	54	0.001	0.010
k__Bacteria.p__Firmicutes.c__Clostridia.o__Clostridiales.f__Christensenellaceae.g__	Body mass index Z-score	-0.753	0.220	77	0.001	0.010
k__Bacteria.p__Actinobacteria.c__Coriobacteriia.o__Coriobacteriales.f__Coriobacteriaceae.g__Collinsella	Body mass index Z-score	0.332	0.118	127	0.005	0.050
k__Bacteria.p__Firmicutes.c__Clostridia.o__Clostridiales.f__.g__	Body mass index Z-score	-0.311	0.110	131	0.005	0.050
k__Bacteria.p__Firmicutes.c__Erysipelotrichi.o__Erysipelotrichales.f__Erysipelotrichaceae.g__Bulleidia	Body mass index Z-score	-0.734	0.273	46	0.007	0.070
k__Bacteria.p__Bacteroidetes.c__Bacteroidia.o__Bacteroidales.f__.Odoribacteraceae.g__Odoribacter	Body mass index Z-score	-0.562	0.210	53	0.007	0.071
k__Bacteria.p__Firmicutes.c__Erysipelotrichi.o__Erysipelotrichales.f__Erysipelotrichaceae.g__Catenibacterium	Carbohydrates intake (% Kcal)	1.491	0.046	58	0.000	0.000
k__Bacteria.p__Firmicutes.c__Clostridia.o__Clostridiales.f__Clostridiaceae.g__Clostridium sensu stricto	Carbohydrates intake (% Kcal)	0.628	0.051	40	0.000	0.000
k__Bacteria.p__Proteobacteria.c__Gammaproteobacteria.o__Pasteurellales.f__Pasteurellaceae	Carbohydrates intake (% Kcal)	4.792	1.543	57	0.002	0.025
k__Bacteria.p__Firmicutes.c__Bacilli.o__Lactobacillales.f__Streptococcaceae.g__Lactococcus	Carbohydrates intake (% Kcal)	4.946	1.626	47	0.002	0.030

k__Bacteria.p__Firmicutes.c__Clostridia.o__Clostridiales.f__Lachnospiraceae.g__Ruminococcus	Carbohydrates intake (% Kcal)	-4.880	1.646	49	0.003	0.036
k__Bacteria.p__Firmicutes.c__Clostridia.o__Clostridiales.f__Lachnospiraceae.g__Lachnospira	Carbohydrates intake (% Kcal)	1.959	0.736	125	0.008	0.072
k__Bacteria.p__Firmicutes.c__Erysipelotrichi.o__Erysipelotrichales.f__Erysipelotrichaceae.g__Clostridium	Carbohydrates intake (% Kcal)	-4.379	1.718	34	0.011	0.089
k__Bacteria.p__Firmicutes.c__Erysipelotrichi.o__Erysipelotrichales.f__Erysipelotrichaceae.g__Catenibacterium	Protein intake (% Kcal)	0.995	0.026	58	0.000	0.000
k__Bacteria.p__Firmicutes.c__Clostridia.o__Clostridiales.f__Clostridiaceae.g__Clostridium sensu stricto	Protein intake (% Kcal)	0.668	0.029	40	0.000	0.000
k__Bacteria.p__Firmicutes.c__Bacilli.o__Lactobacillales.f__Streptococcaceae.g__Lactococcus	Protein intake (% Kcal)	3.053	0.945	47	0.001	0.018
k__Bacteria.p__Firmicutes.c__Clostridia.o__Clostridiales.f__Lachnospiraceae.g__Ruminococcus	Protein intake (% Kcal)	-3.081	0.961	49	0.001	0.018
k__Bacteria.p__Proteobacteria.c__Gammaproteobacteria.o__Pasteurellales.f__Pasteurellaceae	Protein intake (% Kcal)	2.730	0.898	57	0.002	0.030
k__Bacteria.p__Firmicutes.c__Erysipelotrichi.o__Erysipelotrichales.f__Erysipelotrichaceae.g__Clostridium	Protein intake (% Kcal)	-2.730	1.008	34	0.007	0.068
k__Bacteria.p__Firmicutes.c__Erysipelotrichi.o__Erysipelotrichales.f__Erysipelotrichaceae.g__Catenibacterium	Fat intake (% Kcal)	0.709	0.035	58	0.000	0.000
k__Bacteria.p__Actinobacteria.c__Coriobacteriia.o__Coriobacteriales.f__Coriobacteriaceae.__	Fat intake (% Kcal)	-0.460	0.098	51	0.000	0.000
k__Bacteria.p__Proteobacteria.c__Betaproteobacteria.o__Burkholderiales.f__Comamonadaceae.g__Delftia	Fat intake (% Kcal)	2.419	0.802	43	0.003	0.031
k__Bacteria.p__Firmicutes.c__Clostridia.o__Clostridiales.f__Clostridiaceae.g__Clostridium sensu stricto	Fat intake (% Kcal)	-0.129	0.043	40	0.003	0.033
k__Bacteria.p__Proteobacteria.c__Gammaproteobacteria.o__Pasteurellales.f__Pasteurellaceae	Fat intake (% Kcal)	3.516	1.227	57	0.004	0.047
k__Bacteria.p__Bacteroidetes.c__Bacteroidia.o__Bacteroidales.f__Barnesiellaceae.g__	Fat intake (% Kcal)	2.730	1.064	62	0.010	0.085
k__Bacteria.p__Firmicutes.c__Clostridia.o__Clostridiales.f__Lachnospiraceae.g__Ruminococcus	Fat intake (% Kcal)	-3.387	1.318	49	0.010	0.085
k__Bacteria.p__Firmicutes.c__Clostridia.o__Clostridiales.f__Clostridiaceae.g__Clostridium sensu stricto	Saturated fatty acids intake (% Kcal)	0.448	0.017	40	0.000	0.000
k__Bacteria.p__Actinobacteria.c__Coriobacteriia.o__Coriobacteriales.f__Coriobacteriaceae.__	Saturated fatty acids intake (% Kcal)	0.268	0.038	51	0.000	0.000
k__Bacteria.p__Firmicutes.c__Erysipelotrichi.o__Erysipelotrichales.f__Erysipelotrichaceae.g__Catenibacterium	Saturated fatty acids intake (% Kcal)	0.045	0.013	58	0.000	0.008
k__Bacteria.p__Bacteroidetes.c__Bacteroidia.o__Bacteroidales.f__Barnesiellaceae.g__	Saturated fatty acids intake (% Kcal)	-1.093	0.403	62	0.007	0.068
k__Bacteria.p__Firmicutes.c__Clostridia.o__Clostridiales.f__.Mogibacteriaceae..g__Mogibacterium	Saturated fatty acids intake (% Kcal)	1.205	0.457	53	0.008	0.075
k__Bacteria.p__Bacteroidetes.c__Bacteroidia.o__Bacteroidales.f__Bacteroidaceae.g__Bacteroides	Saturated fatty acids intake (% Kcal)	-0.549	0.214	134	0.010	0.085
k__Bacteria.p__Firmicutes.c__Clostridia.o__Clostridiales.f__Clostridiaceae.g__Clostridium sensu stricto	Poly unsaturated fatty acids intake (% Kcal)	0.305	0.012	40	0.000	0.000
k__Bacteria.p__Firmicutes.c__Erysipelotrichi.o__Erysipelotrichales.f__Erysipelotrichaceae.g__Catenibacterium	Poly unsaturated fatty acids intake (% Kcal)	-0.120	0.011	58	0.000	0.000
k__Bacteria.p__Proteobacteria.c__Betaproteobacteria.o__Burkholderiales.f__Comamonadaceae.g__Delftia	Poly unsaturated fatty acids intake (% Kcal)	-3.174	0.365	43	0.000	0.000
k__Bacteria.p__Actinobacteria.c__Coriobacteriia.o__Coriobacteriales.f__Coriobacteriaceae.__	Poly unsaturated fatty acids intake (% Kcal)	0.120	0.027	51	0.000	0.000
k__Bacteria.p__Bacteroidetes.c__Bacteroidia.o__Bacteroidales.f__Barnesiellaceae.g__	Poly unsaturated fatty acids intake (% Kcal)	-1.268	0.315	62	0.000	0.001
k__Bacteria.p__Firmicutes.c__Clostridia.o__Clostridiales.f__Lachnospiraceae.g__Lachnobacterium	Poly unsaturated fatty acids intake (% Kcal)	1.714	0.456	33	0.000	0.004
k__Bacteria.p__Firmicutes.c__Clostridia.o__Clostridiales.f__.Mogibacteriaceae..g__Mogibacterium	Poly unsaturated fatty acids intake (% Kcal)	0.886	0.342	53	0.010	0.085
k__Bacteria.p__Firmicutes.c__Erysipelotrichi.o__Erysipelotrichales.f__Erysipelotrichaceae.g__Catenibacterium	Mono unsaturated fatty acids intake (% Kcal)	0.308	0.013	58	0.000	0.000
d__Bacteria;p__Firmicutes;c__Clostridia;o__Clostridiales;f__Clostridiaceae;g__Clostridium sensu stricto	Mono unsaturated fatty acids intake (% Kcal)	-0.375	0.017	40	0.000	0.000
k__Bacteria.p__Bacteroidetes.c__Bacteroidia.o__Bacteroidales.f__Barnesiellaceae.g__	Mono unsaturated fatty acids intake (% Kcal)	-1.354	0.417	62	0.001	0.017
k__Bacteria.p__Firmicutes.c__Clostridia.o__Clostridiales.f__Lachnospiraceae.g__Lachnospira	Mono unsaturated fatty acids intake (% Kcal)	0.612	0.226	125	0.007	0.069
k__Bacteria.p__Actinobacteria.c__Coriobacteriia.o__Coriobacteriales.f__Coriobacteriaceae.__	Mono unsaturated fatty acids intake (% Kcal)	-0.104	0.039	51	0.008	0.074
k__Bacteria.p__Firmicutes.c__Clostridia.o__Clostridiales.f__Christensenellaceae.g__	Mono unsaturated fatty acids intake (% Kcal)	-1.012	0.392	77	0.010	0.085

Supplementary Table S3. Bacterial composition in the low SES group.

	Unweighted UniFrac					Weighted UniFrac				
	**Df**	**SumofSqrs**	**R2**	**F**	**p-value**	**Df**	**SumofSqrs**	**R2**	**F**	**p-value**
Body mass index Z-score	2	0.430	0.041	1.405	0.046	2	0.079	0.061	2.175	0.023
Fat intake (% Kcal)	1	0.199	0.019	1.297	0.126	1	0.008	0.006	0.450	0.848
Poly unsaturated fatty acids intake (% Kcal)	1	0.167	0.016	1.091	0.344	1	0.027	0.021	1.493	0.181
Mono unsaturated fatty acids intake (% Kcal)	1	0.118	0.011	0.771	0.819	1	0.017	0.013	0.947	0.442
Saturated fatty acids intake (% Kcal)	1	0.133	0.012	0.865	0.651	1	0.017	0.013	0.917	0.435
Protein intake (% Kcal)	1	0.233	0.022	1.521	0.049	1	0.041	0.032	2.262	0.071
Carbohydrates intake (% Kcal)	1	0.135	0.013	0.883	0.639	1	0.014	0.010	0.745	0.571
Residual	60	9.189	0.867	NA	NA	60	1.091	0.843	NA	NA
Total	68	10.603	1.000	NA	NA	68	1.293	1.000	NA	NA

Adonis2 was implemented, with 1,000 permutations.

Supplementary Table S4. Maaslin2 results for the low SES group.

feature	metadata	coef	stderr	N	N.not.0	pval	qval
k__Bacteria.p__Bacteroidetes.c__Bacteroidia.o__Bacteroidales.f__.Paraprevotellaceae..g__	Body mass index Z-score	-0.673	0.052	70	18	0.000	0.000
k__Bacteria.p__Firmicutes.c__Clostridia.o__Clostridiales.f__.Mogibacteriaceae..g__Mogibacterium	Body mass index Z-score	-0.113	0.026	70	31	0.000	0.000
k__Bacteria.p__Actinobacteria.c__Coriobacteriia.o__Coriobacteriales.f__Coriobacteriaceae.g__Atopobium parvulum	Body mass index Z-score	0.896	0.302	70	25	0.003	0.032
k__Bacteria.p__Firmicutes.c__Clostridia.o__Clostridiales.f__.Mogibacteriaceae..g__	Body mass index Z-score	0.307	0.110	70	67	0.005	0.047
k__Bacteria.p__Bacteroidetes.c__Bacteroidia.o__Bacteroidales.f__.Odoribacteraceae..g__Odoribacter	Body mass index Z-score	-0.794	0.307	70	21	0.010	0.077
k__Bacteria.p__Firmicutes.c__Clostridia.o__Clostridiales.f__.Mogibacteriaceae..g__Mogibacterium	Carbohydrates intake (% Kcal)	1.850	0.169	70	31	0.000	0.000
k__Bacteria.p__Bacteroidetes.c__Bacteroidia.o__Bacteroidales.f__.Paraprevotellaceae..g__	Carbohydrates intake (% Kcal)	2.926	0.269	70	18	0.000	0.000
k__Bacteria.p__Actinobacteria.c__Coriobacteriia.o__Coriobacteriales.f__Coriobacteriaceae.__	Carbohydrates intake (% Kcal)	-1.535	0.143	70	33	0.000	0.000
k__Bacteria.p__Bacteroidetes.c__Bacteroidia.o__Bacteroidales.f__.Paraprevotellaceae..g__	Protein intake (% Kcal)	2.080	0.169	70	18	0.000	0.000
k__Bacteria.p__Actinobacteria.c__Coriobacteriia.o__Coriobacteriales.f__Coriobacteriaceae.__	Protein intake (% Kcal)	-1.083	0.094	70	33	0.000	0.000
k__Bacteria.p__Firmicutes.c__Clostridia.o__Clostridiales.f__.Mogibacteriaceae..g__Mogibacterium	Protein intake (% Kcal)	1.059	0.105	70	31	0.000	0.000
k__Bacteria.p__Bacteroidetes.c__Bacteroidia.o__Bacteroidales.f__.Paraprevotellaceae..g__	Fat intake (% Kcal)	2.533	0.155	70	18	0.000	0.000
k__Bacteria.p__Actinobacteria.c__Coriobacteriia.o__Coriobacteriales.f__Coriobacteriaceae.__	Fat intake (% Kcal)	-1.126	0.102	70	33	0.000	0.000
k__Bacteria.p__Firmicutes.c__Clostridia.o__Clostridiales.f__.Mogibacteriaceae..g__Mogibacterium	Fat intake (% Kcal)	0.912	0.123	70	31	0.000	0.000
k__Bacteria.p__Firmicutes.c__Clostridia.o__Clostridiales.f__.Mogibacteriaceae..g__Mogibacterium	Saturated fatty acids intake (% Kcal)	-0.216	0.043	70	31	0.000	0.000
k__Bacteria.p__Bacteroidetes.c__Bacteroidia.o__Bacteroidales.f__Bacteroidaceae.g__Bacteroides	Saturated fatty acids intake (% Kcal)	-0.817	0.297	70	67	0.006	0.050
k__Bacteria.p__Bacteroidetes.c__Bacteroidia.o__Bacteroidales.f__.Paraprevotellaceae..g__	Poly unsaturated fatty acids intake (% Kcal)	-0.355	0.055	70	18	0.000	0.000
k__Bacteria.p__Firmicutes.c__Clostridia.o__Clostridiales.f__.Mogibacteriaceae..g__Mogibacterium	Poly unsaturated fatty acids intake (% Kcal)	0.129	0.033	70	31	0.000	0.001
k__Bacteria.p__Bacteroidetes.c__Bacteroidia.o__Bacteroidales.f__.Odoribacteraceae..g__Odoribacter	Poly unsaturated fatty acids intake (% Kcal)	-1.373	0.437	70	21	0.002	0.020
k__Bacteria.p__Bacteroidetes.c__Bacteroidia.o__Bacteroidales.f__Bacteroidaceae.g__Bacteroides	Poly unsaturated fatty acids intake (% Kcal)	-0.665	0.227	70	67	0.003	0.035
k__Bacteria.p__Bacteroidetes.c__Bacteroidia.o__Bacteroidales.f__Rikenellaceae.__	Poly unsaturated fatty acids intake (% Kcal)	-1.399	0.483	70	18	0.004	0.036
k__Bacteria.p__Firmicutes.c__Bacilli.o__Lactobacillales.f__Lactobacillaceae.g__Lactobacillus	Poly unsaturated fatty acids intake (% Kcal)	0.664	0.232	70	66	0.004	0.039

k__Bacteria.p__Actinobacteria.c__Coriobacteriia.o__Coriobacteriales.f__Coriobacteriaceae.g__Atopobium parvulum	Poly unsaturated fatty acids intake (% Kcal)	0.895	0.344	70	25	0.009	0.076
k__Bacteria.p__Bacteroidetes.c__Bacteroidia.o__Bacteroidales.f__.Paraprevotellaceae..g__	Mono unsaturated fatty acids intake (% Kcal)	-0.392	0.089	70	18	0.000	0.000
k__Bacteria.p__Actinobacteria.c__Coriobacteriia.o__Coriobacteriales.f__Coriobacteriaceae.__	Mono unsaturated fatty acids intake (% Kcal)	0.166	0.046	70	33	0.000	0.004
k__Bacteria.p__Actinobacteria.c__Coriobacteriia.o__Coriobacteriales.f__Coriobacteriaceae.g__Atopobium parvulum	Mono unsaturated fatty acids intake (% Kcal)	1.522	0.503	70	25	0.002	0.028

Supplementary Table S5. Bacterial composition in the high SES group.

	Unweighted UniFrac					Weighted UniFrac				
	**Df**	**SumofSqrs**	**R2**	**F**	**p-value**	**Df**	**SumofSqrs**	**R2**	**F**	**p-value**
Body mass index Z-score	1	0.225	0.018	1.290	0.131	1	0.035	0.024	1.616	0.162
Fat intake (% Kcal)	1	0.189	0.015	1.083	0.332	1	0.003	0.002	0.139	0.989
Poly unsaturated fatty acids intake (% Kcal)	1	0.463	0.037	2.653	0.001	1	0.029	0.020	1.331	0.217
Mono unsaturated fatty acids intake (% Kcal)	1	0.216	0.017	1.236	0.181	1	0.006	0.004	0.258	0.957
Saturated fatty acids intake (% Kcal)	1	0.231	0.018	1.325	0.121	1	0.029	0.020	1.334	0.225
Protein intake (% Kcal)	1	0.196	0.016	1.123	0.275	1	0.009	0.006	0.408	0.848
Carbohydrates intake (% Kcal)	1	0.170	0.014	0.977	0.443	1	0.013	0.009	0.588	0.711
Residual	62	10.813	0.865	NA	NA	62	1.335	0.916	NA	NA
Total	69	12.502	1.000	NA	NA	69	1.457	1.000	NA	NA

Adonis2 was implemented, with 1,000 permutations.

Supplementary Table S6. Maaslin2 results for the high SES group.

feature	metadata	coef	stderr	N	N.not.0	pval	qval
k__Bacteria.p__Firmicutes.c__Clostridia.o__Clostridiales.f__Lachnospiraceae.__	Body mass index Z-score	0.390	0.101	69	69	0.000	0.014
k__Bacteria.p__Firmicutes.c__Bacilli.o__Turicibacterales.f__Turicibacteraceae.g__Turicibacter	Body mass index Z-score	-0.451	0.157	69	63	0.004	0.073
k__Bacteria.p__Bacteroidetes.c__Bacteroidia.o__Bacteroidales.f__Porphyromonadaceae.g__Barnesiella	Carbohydrates intake (% Kcal)	-7.862	2.375	69	19	0.001	0.035
k__Bacteria.p__Firmicutes.c__Bacilli.o__Lactobacillales.f__Streptococcaceae.g__Lactococcus	Carbohydrates intake (% Kcal)	7.354	2.355	69	27	0.002	0.039
k__Bacteria.p__Bacteroidetes.c__Bacteroidia.o__Bacteroidales.f__Porphyromonadaceae.g__Barnesiella	Protein intake (% Kcal)	-4.273	1.304	69	19	0.001	0.035
k__Bacteria.p__Firmicutes.c__Bacilli.o__Lactobacillales.f__Streptococcaceae.g__Lactococcus	Protein intake (% Kcal)	3.988	1.241	69	27	0.001	0.035
k__Bacteria.p__Bacteroidetes.c__Bacteroidia.o__Bacteroidales.f__Porphyromonadaceae.g__Barnesiella	Fat intake (% Kcal)	-8.109	2.215	69	19	0.000	0.015
k__Bacteria.p__Firmicutes.c__Clostridia.o__Clostridiales.f__Lachnospiraceae.g__Lachnobacterium	Poly unsaturated fatty acids intake (% Kcal)	2.378	0.618	69	18	0.000	0.014
k__Bacteria.p__Bacteroidetes.c__Bacteroidia.o__Bacteroidales.f__Porphyromonadaceae.g__Barnesiella	Poly unsaturated fatty acids intake (% Kcal)	1.593	0.500	69	19	0.001	0.035
k__Bacteria.p__Firmicutes.c__Bacilli.o__Lactobacillales.f__Carnobacteriaceae.__	Poly unsaturated fatty acids intake (% Kcal)	1.230	0.386	69	24	0.001	0.035
k__Bacteria.p__Bacteroidetes.c__Bacteroidia.o__Bacteroidales.f__.Paraprevotellaceae..g__Paraprevotella	Mono unsaturated fatty acids intake (% Kcal)	2.790	0.969	69	18	0.004	0.073

Supplementary Table S7. Maaslin2 results for the participants with obesity.

feature	metadata	coef	stderr	N	N.not.0	pval	qval
k__Bacteria.p__Bacteroidetes.c__Bacteroidia.o__Bacteroidales.f__.Paraprevotellaceae..g__	Fat intake (% Kcal)	0.511	0.167	79	16	0.002	0.064
k__Bacteria.p__Bacteroidetes.c__Bacteroidia.o__Bacteroidales.f__.Barnesiellaceae..g__	Poly unsaturated fatty acids intake (% Kcal)	-1.450	0.413	79	33	0.000	0.029
k__Bacteria.p__Firmicutes.c__Clostridia.o__Clostridiales.f__.Mogibacteriaceae..g__Mogibacterium	Poly unsaturated fatty acids intake (% Kcal)	1.369	0.436	79	33	0.002	0.064
k__Bacteria.p__Bacteroidetes.c__Bacteroidia.o__Bacteroidales.f__.Barnesiellaceae..g__	Saturated fatty acids intake (% Kcal)	-2.300	0.570	79	33	0.000	0.005
k__Bacteria.p__Bacteroidetes.c__Bacteroidia.o__Bacteroidales.f__.Paraprevotellaceae..g__	Mono unsaturated fatty acids intake (% Kcal)	-0.469	0.094	79	16	0.000	0.000
k__Bacteria.p__Firmicutes.c__Clostridia.o__Clostridiales.f__Ruminococcaceae.g__Ruminococcus	Mono unsaturated fatty acids intake (% Kcal)	-0.611	0.189	79	79	0.001	0.060
k__Bacteria.p__Firmicutes.c__Clostridia.o__Clostridiales.f__.Mogibacteriaceae..g__Mogibacterium	Mono unsaturated fatty acids intake (% Kcal)	1.908	0.627	79	33	0.002	0.064
